# Genome‑wide identification of the NHX gene family in *Forsythia* identifies *FsNHX3* and *FsNHX6* as potential candidates involved in abiotic stress responses

**DOI:** 10.1371/journal.pone.0359565

**Published:** 2026-10-01

**Authors:** Lulu Li, Xiaojun Ma, Aiqin Ding, Guanning Zhang, Jiahui Zhou, Congcong Liu, Chunli Chen, Xiaohan Tang, Xuexiang Li, Jing Shu, Xinhong Wang

**Affiliations:** 1 College of Forestry Engineering, Shandong Agriculture and Engineering University, Jinan, P R China; 2 College of Agricultural Science and Technology, Shandong Agriculture and Engineering University, Jinan, P R China; Arish university, Faculty of agricultural and environmental sciences, EGYPT

## Abstract

The Na ⁺ /H⁺ antiporter (NHX) gene family plays a central role in maintaining cellular ion homeostasis and responding to abiotic stress in plants. However, the systematic identification and functional characterization of this gene family in woody plants of the genus *Forsythia* remain unexplored. To address this, genome-wide identification and systematic comparative analysis of the NHX gene family were performed in three *Forsythia* species, *Forsythia suspensa*, *F. viridissima*, and *F. ovata*, with a focus on elucidating *F. suspensa* NHX gene expression under various types of stress: salt, drought (PEG-simulated), and exogenous abscisic acid (ABA). In total, 18 NHX members were identified and were classified into three subclasses based on phylogenetic analysis, resulting in 12 vacuolar membrane-localized ‘Vac’-class members, four plasma-membrane-localized ‘PM’-class members, and two endosomal system-localized ‘Endo’-class members. Those proteins within the same evolutionary clade exhibit highly conserved structural features in terms of motif arrangement and gene structure. Synteny analysis identified whole-genome or segmental duplication as the primary driving force for the expansion of this family, with all of the duplicated gene pairs having undergone strong purifying selection. Based on interspecies synteny analysis, *F. suspensa* and *F. viridissima* exhibited the highest degree of NHX gene family homology. Expression analysis revealed that the *F. suspensa* NHX gene family exhibits distinct and tissue-specific responses to drought, salt, and exogenous ABA stress. Among the NHX members, FsNHX3 and FsNHX6 exhibited the most pronounced and sustained transcriptional upregulation across the three stress treatments, suggesting their potential involvement in stress response. These findings provide valuable insights for the further exploration of the functions and molecular mechanisms of the NHX gene family in *Forsythia* under salt and drought stress, offering valuable candidate genetic resources for breeding *Forsythia* varieties with enhanced tolerance to salinity and dehydration.

## 1 Introduction

Soil salinization and drought represent increasing challenges affecting agricultural productivity and ecosystem sustainability globally [1]. When extracellular Na⁺ concentrations are high, Na⁺ enters plant cells, leading to cytoplasmic accumulation of Na⁺. High Na^+^ levels can lead to osmotic stress, ionic toxicity, and oxidative damage, ultimately affecting plant growth, development, yield, and quality [2]. Drought reduces the soil water potential, ultimately reducing the ability of the roots to take up water, leading to cellular dehydration, osmotic stress, and ion imbalance. *Forsythia* species, belonging to the Oleaceae family, are important early-spring-flowering shrubs that are widely distributed in East Asia and that possess significant ornamental, medicinal, and ecological value. *Forsythia suspensa*, a traditional Chinese medicinal plant, produces dried fruits rich in lignan compounds such as forsythin and forsythiaside A, which exhibit anti-inflammatory, antioxidant, antibacterial, antiviral, and anti-allergic activities [3–5]. However, in recent years, the cultivation and production of *Forsythia* species have been increasingly affected globally by drought and soil salinization.

Na⁺/H⁺ antiporter (NHX) family members are key regulators of intracellular ion homeostasis in plants. NHX members synergistically regulate Na⁺ and K⁺ balance and compartmentalization at the cellular, tissue, and whole-plant levels, forming a critical network that enables adaptation to salt stress and other adverse conditions while maintaining normal growth and development [6]. NHX proteins, which belong to the monovalent cation/proton antiporter 1 superfamily, are transmembrane proteins of ca. 550 amino acid residues and contain a highly conserved Na⁺/H⁺ exchanger domain [7]. Most NHX proteins consist of 10–12 transmembrane helices and have a hydrophobic N-terminal domain and hydrophilic C-terminal tail [8]. The N-terminal regions of these NHX species exhibit high levels of homology. For example, the third or fourth transmembrane domain contains the conserved sequence FF(I/L)(Y/F)LFLLPPI, which is an amiloride-binding site and a competitive inhibitor binding site for transport activity [9]. In *Arabidopsis thaliana*, eight NHX members (*AtNHX1*–*AtNHX8*) have been identified. The NHX family can be subdivided into three distinct classes based on subcellular localization, cellular function, and phylogenetic relationships: the vacuolar membrane-localized ‘Vac’ class (e.g., *AtNHX1*–*AtNHX4*), the endosomal system-localized ‘Endo’ class (e.g., *AtNHX5* and *AtNHX6*), and the plasma membrane-localized ‘PM’ class (e.g., *AtNHX7/SOS1* and *AtNHX8*) [8,10].

NHX proteins play central and complex roles in maintaining cation homeostasis. They are operated by the transmembrane proton motive force generated by proton pumps like H⁺ -ATPase and H⁺ -PPase that provide the energy for Na⁺/H⁺ or K⁺/H⁺ exchange [6,11]. The cellular role of NHX varies depending on its localization. The plasma membrane-localized members, including *AtNHX7/SOS1*, actively expel excess Na^+^ from the cytoplasm and thus alleviate cytoplasmic Na^+^ toxicity [12,13]. The vacuolar membrane-localized members sequester Na^+^ into the vacuolar lumen, reduce Na^+^ toxicity in the cytoplasm, and utilize vacuolar Na^+^ for osmotic adjustment to maintain cell turgor and enhance salt tolerance [10,14–16]. Recent studies have revealed that, in *Arabidopsis*, NHX1 and NHX2 have a greater affinity for K⁺ than Na⁺ , predominantly pumping K⁺ into the vacuole; this mechanism maintains cell turgidity and helps to maintain pH, thus affecting cell growth, tissue elongation, and stomatal movement [17,18]. The endomembrane-localized members, such as *AtNHX5* and *AtNHX6*, which are localized to the endosome and Golgi apparatus, regulate vesicle trafficking and protein processing by modulating the compartment pH and ion balance [10,19]. Vesicle trafficking and the fusion of vesicles with vacuoles are important aspects of cellular responses to abiotic stress [20,21].

Regulation of intracellular cation homeostasis by NHX proteins underpins their vital roles in plant tolerance to salinity, alkalinity, and drought. Enhancing salt tolerance—the core function of NHX proteins—has constituted both the primary reason for their initial discovery and the basis for the extensive later research [22]. NHX gene overexpression, heterologous or homologous, has been widely shown to substantially enhance salt tolerance in plants. In cotton, the vacuolar-localized *GhNHX1* is significantly activated under salt stress, and its silencing reduces seedling tolerance to high salt concentrations [23]. Both the vacuolar-type *GhNHX3D* and endosomal-type *GhNHX4A* have been shown to enhance the salt tolerance of salt-sensitive yeast mutants, whereas virus-induced gene silencing of these genes in cotton plants reduced salt tolerance [24,25]. In transgenic tomato plants, overexpression of NHX genes such as *LeNHX2* and *LeNHX4*, or of the exogenous *AtNHX1*, significantly enhanced salt tolerance, enabling them to maintain flowering, growth, and fruit yield in high-salinity soils; simultaneously, vacuolar Na⁺ sequestration driven by these genes reduced sodium buildup in the fruit, thereby safeguarding fruit quality against salt‑induced damage [26–28].

Recent studies have demonstrated that NHX proteins can enhance plant drought tolerance. By promoting the compartmentalization of Na⁺ and K⁺ into vacuoles, NHX proteins directly enhance the osmotic adjustment capacity of plants. *Arabidopsis* plants overexpressing the wheat tonoplast *TNHX1* and H⁺ -pyrophosphatase TVP1 exhibited enhanced salt and drought tolerance as well as improved water retention capacity [29]. Under drought stress, transgenic *Arabidopsis* plants overexpressing soybean *GmNHX1* or *GmNHX3* accumulated more Na⁺ and K⁺ ions, thereby reducing their cell water potential, facilitating water uptake from dry soil, and maintaining higher relative water content in the leaves [30]. Under water-deficit conditions, the activity of antioxidant enzymes such as peroxidase and superoxide dismutase was significantly higher in these transgenic plants than in the wild-type plants, whereas their malondialdehyde content was reduced, indicating less oxidative damage to cell membranes [30].

*Forsythia* species have adapted to diverse habitats during their long evolutionary history. For example, *F. suspensa* is widely distributed in China but is more commonly found in warm temperate regions. *Forsythia viridissima* is predominantly distributed in the subtropical regions of China, particularly in the Yangtze River basin [31], and *Forsythia ovata* is native to the Korean Peninsula and cultivated in northeastern China. This divergence in ecological adaptation provides ideal natural material for investigating the molecular basis of stress adaptation in *Forsythia* species.

NHX genes, important transmembrane transporters of Na^+^, K^+^, and H^+^, are central to cellular ionic homeostasis, pH balancing, and plant responses to abiotic stresses such as salt and drought [17,32]. Although NHX genes have been systematically identified and functionally characterized in various models and crop plants, including *Arabidopsis thaliana*, rice, and cotton, their genome-wide identification, evolutionary features, and regulatory mechanisms under stress conditions remain largely unexplored in *Forsythia*.

To address this gap, genome-wide identification and systematic comparative analysis of NHX genes were conducted in three *Forsythia* species: *F. suspensa*, *F. viridissima*, and *F. ovata*. The research objectives were to identify NHX members in these *Forsythia* species and systematically analyze their physicochemical properties, phylogenetic relationships, gene structures, conserved motifs, and predicted subcellular localization. The evolutionary mechanisms and duplication events within this family were examined through intra- and inter-species synteny analysis. Finally, transcriptomic data and quantitative real-time PCR were used to examine NHX gene expression in *F. suspensa* under salt and drought stress and exogenous abscisic acid treatment. This study aimed to elucidate the evolutionary characteristics and potential functions of NHX genes in these *Forsythia* species, to reveal their potential regulatory mechanisms in response to different abiotic stresses, and provide key candidate gene resources for breeding salt- and drought-tolerant Forsythia varieties.

## 2 Materials and methods

### 2.1 Identification of NHX genes

We retrieved eight *AtNHX* sequences from the TAIR database. Whole-genome and annotation data for *F. suspensa* were obtained from the National Genomics Data Center (https://ngdc.cncb.ac.cn/) under accession PRJCA023056 [33]; for *F. ovata*, these data were sourced from the National Center for Biotechnology Information (NCBI) via accession PRJNA1086660 [34]; and for *F. viridissima*, these data were acquired from The Integrated Medicinal Plantomics database [31] (https://www.bic.ac.cn/IMP/#/speciesInfo?ID=494). Using TBtools v2.372 [35], these genome and annotation datasets were filtered to remove fragmented contigs, and the longest transcript per gene was extracted as the basis for subsequent analyses. Using the eight *AtNHX* sequences as queries, BLASTP homology searches were performed against the proteomes of *F. suspensa*, *F. ovata*, and *F. viridissima*, with an E-value threshold of 1 × 10^−5^. Concurrently, the hidden Markov model (HMM) profile of the NHX conserved domain PF00999 was retrieved from the Pfam database and used to screen the three proteomes via HMMER. Candidate NHX genes were defined as those sequences identified by both BLASTP and HMMER searches. To verify that all of the candidate sequences possess the typical Na⁺/H⁺ exchanger domain, they were analyzed using the NCBI CDD and SMART databases under the default parameters.

Publicly available transcriptome data were used to refine the gene models of *FsNHX* members whose Na⁺/H⁺ exchanger domain annotations were incomplete. RNA-seq reads of *F. suspensa* (accessions SRR10766265, SRR10766266, SRR10766267, SRR10829626, and SRR10829647) were retrieved from the NCBI SRA database. Using the ‘Convert SRA to Fastq Files’ utility within TBtools v2.372 [35], the SRA files were transformed into the FASTQ format. The obtained reads were then mapped onto the reference genome, and sorted BAM files were produced using the sort function in SAMtools. To manually refine the exon–intron structure of the annotated target genes, the resulting alignments were visually examined using IGV-GSAman.

### 2.2 Chromosomal localization and physicochemical properties

Based on the *F. suspensa*, *F. ovata*, and *F. viridissima* genome annotation data, the chromosomal location of each NHX gene was extracted using TBtools v2.372 and the resulting positions were visualized for chromosome mapping [35]. The ExPASy ProtParam server (http://web.expasy.org/protparam/) was used to compute the physicochemical properties of the NHX proteins [36]. The subcellular destinations of *FsNHX*, *FvNHX*, and *FoNHX* were predicted using the Plant-mPLoc algorithm (http://www.csbio.sjtu.edu.cn/bioinf/plant-multi/) [37]. Transmembrane helix regions were identified using TMHMM Server v2.0 (https://services.healthtech.dtu.dk/services/TMHMM-2.0/). Secondary structural elements were characterized using the SOPMA web server (https://npsa.lyon.inserm.fr/cgi-bin/npsa_automat.pl?page=/NPSA/npsa_sopma.html), while three-dimensional models were generated using AlphaFold3 (https://deepmind.google/technologies/alphafold/alphafold-server/) [38].

### 2.3 Phylogenetic analysis

Multiple sequence alignment of the NHX proteins was performed using the MUSCLE algorithm integrated in MEGA11, applying the default parameters [39]. For the subsequent phylogenetic reconstruction, NHX protein sequences from ten plant species, namely *F. suspensa*, *F. ovata*, *F. viridissima*, *A. thaliana*, *Populus euphratica*, *Triticum aestivum*, *Oryza sativa*, *Solanum lycopersicum*, *Punica granatum*, and *Vitis vinifera*, were included. Maximum likelihood (ML) inference was performed under the JTT + G + F model, which combines the Jones–Taylor–Thornton substitution matrix with empirical amino acid frequencies and incorporates a gamma distribution along with a proportion of invariant sites to account for rate heterogeneity across sites. Branch support was evaluated via nonparametric bootstrap analysis with 1,000 replicates. The resulting phylogenetic tree was visualized and annotated using the iTOL web server (https://itol.embl.de).

### 2.4 Conserved motifs, gene structure, and multiple sequence alignment

Conserved motifs in the NHX proteins were predicted using the MEME online tool (https://meme-suite.org/meme/tools/meme), with the maximum motif number increased from the default of 3–10 [40]. To determine the exon–intron architecture of the NHX genes, their coding sequences (CDS) were aligned with the corresponding genomic DNA sequences using TBtools v2.372 [35]. Multiple sequence alignment was performed using the Clustalw online tool (https://www.genome.jp/tools-bin/clustalw), using the default settings, to assess conservation of functional domains. The resulting multiple sequence alignments were visualized using ESPript v3.2 [41].

### 2.5 Synteny and selection pressure analysis

Intra- and inter-species synteny analyses of *FsNHX*, *FoNHX*, and *FvNHX* were performed using the McScanX plugin of TBtools, with an E-value threshold of ≤1.0E-10 [35]. TBtools was used to compute the *K*_*a*_, *K*_*s*_, and *K*_*a*_/*K*_*s*_ ratios for the syntenic gene pairs [35]. Divergence times were then calculated as *T* = *K*_*s*_ / 2*r* × 10^−6^ million years ago (Mya), where *r* represents the synonymous substitution rate of 8.83 × 10^−9^ substitutions per site per year for *Fraxinus* species [42].

### 2.6 Cis-acting element analysis

For each NHX gene, 2 kb sequences upstream of the transcription start site were retrieved from the genome assemblies of *F. suspensa*, *F. viridissima*, and *F. ovata* using TBtools v2.372 [35]. The PlantCARE database (http://bioinformatics.psb.ugent.be/webtools/plantcare/html/) was then used to identify cis‑acting regulatory elements (CAREs) within these promoter sequences [43]. The identified CAREs encompassing motifs related to stress, hormones, development, and light responses were visualized using GraphPad Prism 9.

### 2.7 NHX gene expression under drought and low temperature stress based on RNA-Seq data

Transcriptome data for four populations of *F. suspensa* under drought stress (accession numbers SRR10829625 to SRR10829648) [44] and cold stress (accession numbers SRR10766265 to SRR10766288) [45] were obtained from the NCBI Sequence Read Archive (SRA) database. The four populations were from Wuzhi Mountain, Hebei (HBWZ); Wulaofeng, Shanxi (SXWL); Huashan, Shaanxi (SXHM); and Laojun Mountain, Shaanxi (SXLJ). For the drought-treatment group, samples were collected from leaves of six-month-old *F. suspensa* seedlings subjected for 3 h to soil water contents of 80% (control) or 20%. For the cold-treatment group, samples were collected from the leaves of six-month-old *F. suspensa* seedlings subjected for one day to a cultivation temperature of 25 °C (control) or 4 °C.

The SRA Toolkit v2.10.9 was used to retrieve RNA-seq datasets from the NCBI SRA database. For every sample, the corresponding SRA file was fetched using the prefetch command, followed by conversion into FASTQ format using fastq-dump (or fasterq-dump) with the --split-files flag to separate paired‑end reads. Raw sequencing reads were subjected to quality control and preprocessing using Fastp v0.23.2 [46] with the default parameters. Adapter sequences were removed and bases with Phred quality scores below Q20 were eliminated, and after adapter trimming, reads shorter than 36 bp were discarded. An index for transcriptome quantification was constructed using RSEM v1.3.3 [47] in conjunction with the Bowtie2 alignment tool v2.4.4 [48]. The reference genomes and their associated gene annotation files in GTF format were downloaded [33]. Index generation was performed by executing the rsem-prepare-reference command with the --gtf flag, using the reference genome FASTA file. For each sample, the filtered paired‑end reads were mapped to the reference genome and simultaneously quantified using the rsem-calculate-expression command, which was run using the following arguments: --paired-end, --bowtie2, --estimate-rspd, --append-names, and -p 8 (eight CPU threads were utilized). The resulting output provides both gene‑ and transcript‑level expression measures, including expected read counts, FPKM, and transcripts per million (TPM). From these results, the TPM values were extracted for subsequent differential expression and functional analyses.

### 2.8 Plant materials and treatments

Seeds of *F. suspensa* were collected from Yuncheng, Shanxi Province, China. Healthy and plump seeds were selected and germinated in a constant-temperature incubator at 30°C for 7 d. The germinated seeds were then sown in seedling trays filled with a vermiculite and nutrient soil mixture (1:1, v/v) and cultivated at 22–25°C under a 16 h light/8 h dark photoperiod. Upon reaching the eight-true-leaf stage, uniformly grown seedlings were transferred to Hoagland’s nutrient solution and acclimatized for one week. For the salt stress treatment, seedlings were exposed to Hoagland’s solution supplemented with 100 mM NaCl. This concentration was chosen based on a preliminary gradient experiment (using concentrations of 50, 100, and 200 mM NaCl), in which 100 mM NaCl induced visible stress symptoms within an appropriate timeframe without causing rapid lethality, thus allowing reliable monitoring of transcriptional changes. Leaf and root samples were collected at 0 h (control), 3 h, 6 h, 12 h, 24 h, and 2 d after treatment. For drought stress, Hoagland’s solution containing 20% PEG 6000 was applied, and samples were harvested at 0 h (control), 6 h, 12 h, 24 h, 36 h, 2 d, 4 d, and 8 d. For abscisic acid (ABA) induction, seedlings were placed in Hoagland’s solution with 100 μM ABA, and sampling was performed at 0 h (control), 6 h, 12 h, and 24 h. Three biological replicates were used for each time point, with each replicate comprising three seedlings. The collected samples were immediately frozen in liquid nitrogen and stored at −80°C until further analysis.

### 2.9 RNA extraction and gene expression analysis

To isolate total RNA from each biological replicate, an RNA purification kit (Vazyme, Nanjing, China) was used. RNA concentration and purity were measured using a NanoDrop spectrophotometer (Thermo Fisher Scientific, Waltham, MA, USA). First-strand cDNA was synthesized using the HiScript III RT SuperMix (+gDNA wiper) (Vazyme). The transcript EF1αH was used as an endogenous control for quantitative real time PCR (qRT-PCR). PCR reactions were run on a QuantStudio 5 system (Thermo Fisher Scientific) using the ChamQ Universal SYBR qPCR Master Mix (Vazyme). Three technical replicates were used for each sample. The 2^−ΔΔCt^ method [49] was used to estimate relative transcript levels. Statistical analyses were performed using SPSS 27.0 (IBM, Armonk, NY, USA) and one-way ANOVA and Tukey’s honest significant difference (HSD) post hoc test were used for multiple comparisons. The threshold for significance was set at *p* < 0.05. Plots were generated using GraphPad Prism 9. The primer sequences used here are listed in S1 Table.

## 3 Results

### 3.1 Identification, chromosomal localization, and physicochemical properties of NHX genes in *Forsythia*

In *F. suspensa*, six *FsNHX* genes, distributed across five chromosomes, were identified and were designated *FsNHX1*–*FsNHX6*; in *F. viridissima*, seven *FvNHX* genes, located on six chromosomes, were identified and were designated *FvNHX1*–*FvNHX7*; and in *F. ovata*, five *FoNHX* genes, mapped to three chromosomes, were identified and were designated *FoNHX1*–*FoNHX5* (S2 Table; S1 Fig). In the three *Forsythia* species, a total of 18 NHX proteins were identified, with 482–1,146 amino acids and molecular weights of 53.7–128.3 kDa (Table 1). The isoelectric point (pI) of the different proteins was theoretically predicted to be between 5.43 and 9.07, with eight proteins predicted to be acidic based on their pI (pI < 7) and 10 predicted to be basic. Stability analysis revealed that 13 of the proteins were stable, whereas the other five proteins were unstable. Transmembrane domain prediction revealed that all but two of the NHX proteins have 9–12 transmembrane domains, whereas *FoNHX1* and *FoNHX2* have 6–7 transmembrane domains. Hydrophilicity analysis revealed that 16 of the proteins are mostly hydrophobic, whereas *FoNHX1* and *FoNHX2* are hydrophilic. Based on subcellular localization prediction, 14 of the NHX proteins were predicted to be localized in the vacuoles, whereas *FsNHX2*, *FvNHX3*, *FoNHX1* and *FoNHX2* were predicted to be localized in the plasma membrane. These differences in physicochemical characteristics and subcellular localization suggest that these NHX proteins in *Forsythia* have diverged in terms of function.

**Table 1:** The information and physicochemical properties of NHX family proteins in *Forsythia.*

Gene name	Gene ID	Chr Location	Protein length (aa)	MW (KDa)^1^	pI^2^	Instability Index	GRAVY^3^	Subcellular localization	Number of predicted TMHs^4^
*FsNHX1*	FsusChr2G039080	Chr2: 32642113:32649644	536	59.3	7.71	35.04	0.538	Vacuole	11
*FsNHX2*	FsusChr4G089660	Chr4: 38429392:38480886	1146	128.3	6.06	39.62	0.046	Cell membrane	12
*FsNHX3*	FsusChr11G256370	Chr11: 6126214:6131230	560	62.0	8.18	35.46	0.484	Vacuole	11
*FsNHX4*	FsusChr12G284041.1.gene	Chr12: 2554441:2563484	519	57.9	6.89	41.33	0.578	Vacuole	11
*FsNHX5*	FsusChr12G287400.1.gene	chr12: 6318263:6327983	522	57.8	5.43	44.14	0.427	Vacuole	10
*FsNHX6*	FsusChr13G336770	chr13: 47501952:47511396	566	62.7	8.66	39.82	0.503	Vacuole	10
*FvNHX1*	IMPGFVI1N459	chr1: 3144568:3153227	519	57.8	7.21	42.32	0.597	Vacuole	11
*FvNHX2*	IMPGFVI1N955	chr1: 6846261:68555997	533	59.0	5.43	46.27	0.429	Vacuole	10
*FvNHX3*	IMPGFVI1N13844	Chr2: 27512132:27553481	1144	127.7	6.1	38.74	0.045	Cell membrane	12
*FvNHX4*	IMPGFVI1N41747	Chr5: 46794988:46803411	566	62.7	8.66	38.54	0.498	Vacuole	10
*FvNHX5*	IMPGFVI1N47034	Chr6: 32149939:32156783	546	60.4	7.29	35.24	0.563	Vacuole	11
*FvNHX6*	IMPGFVI1N72049	chr10: 5690707:5695351	560	62.0	8.18	35.06	0.484	Vacuole	11
*FvNHX7*	IMPGFVI1N90390	chr13: 1359352:1362604	528	58.8	6.32	33.02	0.509	Vacuole	9
*FoNHX1*	gene-Fot_02455	chr1: 31820853:31855095	905	102.3	5.99	42.07	−0.137	Cell membrane	6
*FoNHX2*	gene-Fot_02523	chr1: 33052335:33101381	1052	118.2	6.34	39.74	−0.113	Cell membrane	7
*FoNHX3*	gene-Fot_16972	chr1: 4078090:4081240	482	53.7	9.07	31.82	0.532	Vacuole	9
*FoNHX4*	gene-Fot_25093	chr1: 38360649:38367443	536	59.3	7.29	34.99	0.549	Vacuole	10
*FoNHX5*	gene-Fot_25146	chr1: 39478588:39485382	536	59.3	7.29	34.99	0.549	Vacuole	10

^1^MW, molecular weight. ^2^ PI: Isoelectric point. ^3^ GRAVY: grand average of hydropathicity score. ^4^TMH: Transmembrane helices.

**Within these** NHX **proteins,** α-**helices and random coils were identified as the predominant secondary structures, comprising 47.93–56.47% and 29.48–34.85% of the amino acid residues, respectively (**S3 Table**), based on secondary structure analysis. To obtain deeper insights into the structural features of the proteins, their three-dimensional structures were predicted and viewed using AlphaFold3 for homology modeling (**S2 Fig**). The resulting models revealed that these** NHX **members possess a highly conserved structural architecture, providing a structural basis for their functional similarity.**

### 3.2 Phylogenetic analysis

Phylogenetic analysis was performed to elucidate the evolutionary relationships among the NHX family members in these *Forsythia* species and to clarify their orthologous relationships with NHX proteins in other plant species (S4 Table). The full-length NHX protein sequences were aligned, and a maximum likelihood tree was constructed using the full sequences for *F. suspensa*, *F. ovata*, *F. viridissima*, and seven other representative species (Fig 1). The final tree, which clearly separates all of the 92 NHX proteins into the three categories Vac, Endo, and PM (Fig 1), is strongly supported (with a 100% bootstrap value) by the fact that it recapitulates the known classification of the *AtNHX* subfamilies. The Vac clade, which contains four *F. suspensa* proteins, five *F. viridissima* proteins, and three *F. ovata* proteins, was the most abundant clade among the 18 *Forsythia* NHX proteins; the Endo clade, in contrast, is the least abundant, comprising only *FsNHX5* and *FvNHX2*, indicating the possible loss of this subclass in *F. ovata*. The PM clade includes four members, *FoNHX1* and *FoNHX2* from *F. ovata*, one ortholog from *F. suspensa*, and one from *F. viridissima*. Interestingly, the NHX genes of each *Forsythia* species are clustered together within each protein subfamily (Endo, Vac, and PM), revealing their high homology and recent divergence. This phylogenetic framework is consistent with prior findings for *Arabidopsis* and other dicots and is suitable for further functional characterization.

**Fig 1:**
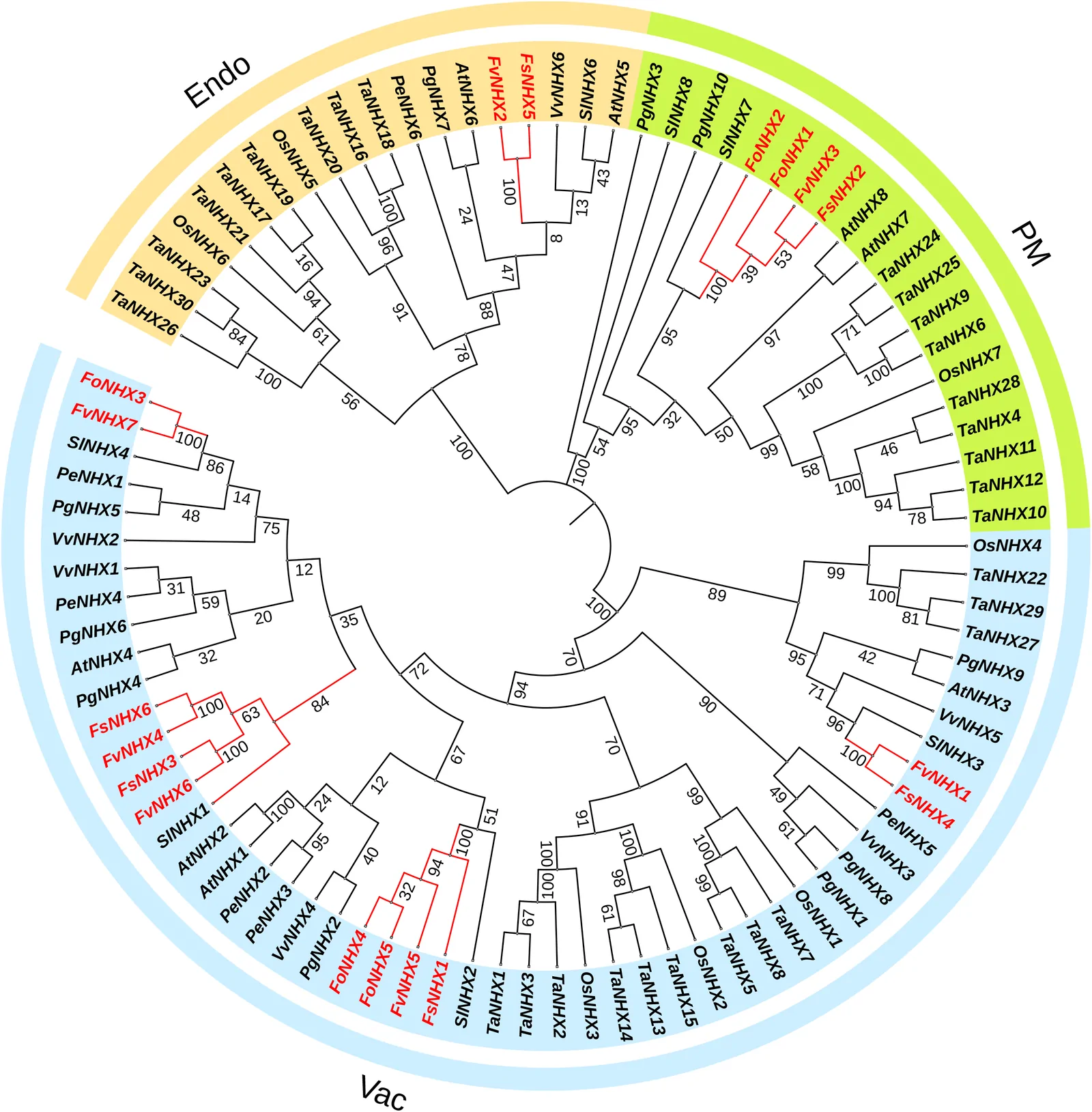
The phylogenetic tree of the NHX gene family. Vacuolar class (Vac), endosomal class (Endo), and plasma membrane class (PM) are marked with blue, yellow, green background bands, respectively. NHX members in *Forsythia* are highlighted in red font. The numbers at the nodes indicate bootstrap 1000.

### 3.3 Conserved domains, motifs, and gene structures

The conserved motifs and gene structures of the 18 NHX members were systematically analyzed to further characterize this gene family in *Forsythia*. A complete Na⁺/H⁺ exchanger domain (PF00999) was found in all 18 *Forsythia* NHX proteins, based on conserved domain analysis (S3 Fig). MEME motif analysis identified ten conserved motifs, and the distribution of these motifs reveals significant protein-subfamily specificity (Fig 2B). Motifs 1, 5, 6, 9, and 10 are common to all three subfamilies. Motifs 3 and 4 are exclusive to the Vac subfamily, and motif 7 is common to the Vac and Endo subfamilies. Based on structural analysis, the genes within each same subfamily exhibit substantial structural similarity (Fig 2C). The Vac subfamily members have 12–14 exons, and all of the Endo subfamily members have 22 exons. In the PM subfamily, *FsNHX2* and *FvNHX4* each have 23 exons, whereas *FoNHX1* and *FoNHX2* have 17 and 22 exons, respectively.

**Fig 2:**
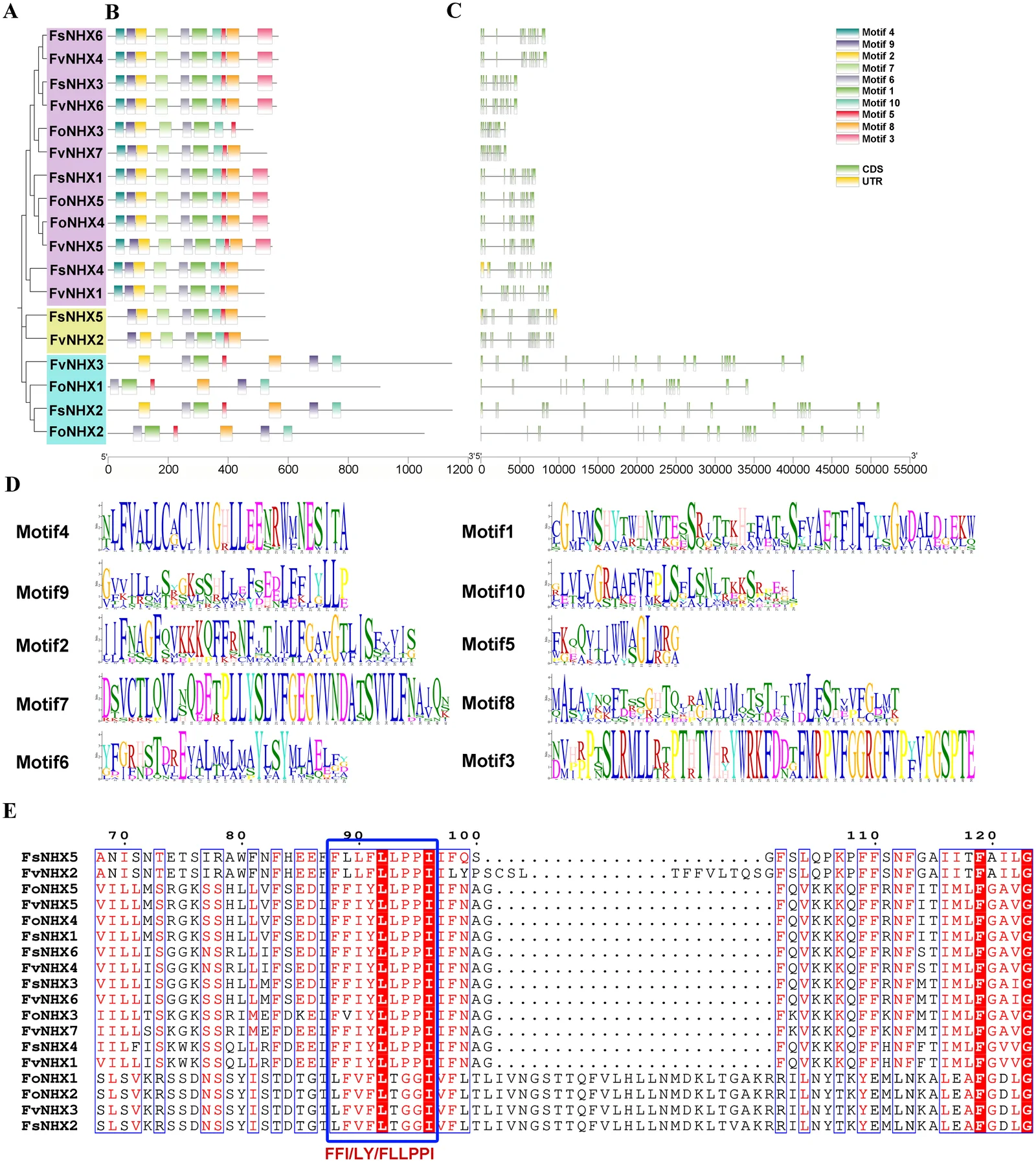
The phylogenetic relationships, conserved motifs, gene structural features and protein sequence alignment of NHX. **(A)** A phylogenetic tree showing relationships of NHX gene family members: Purple: Vac; Yellow: Endo, and Cyan: PM. **(B)** NHX proteins distribution of conserved motifs (motif 1-10: shown by different colored boxes). **(C)** Exon-intron structures of NHX genes. Green: CDS, Yellow: UTRs, Black lines: introns. **(D)** Sequence logos of the conserved motifs found in NHX proteins. **(E)** Multiple sequence alignment of amiloride-binding site (FFI/LY/FLLPPI) among NHX. The conserved region “FFI/LY/FLLPPI” is represented by blue box.

Multiple sequence alignment (Fig 2E) revealed that the amiloride-binding site, FF(I/L)(Y/F)LFLLPPI, is highly conserved in all of the Vac subfamily proteins except for FoNHX3, which has one amino acid substitution (F to V), creating the sequence FVIYLLPPI. Interestingly, this binding site also occurs in two of the Endo subfamily NHX proteins, although both of these have a single amino acid substitution, and the Endo subfamily may retain functional characteristics similar to those of the Vac subfamily.

### 3.4 Duplication events, syntenic relationships, and selective pressure

To investigate the evolutionary mechanisms of the NHX gene family in *F. suspensa*, *F. viridissima*, and *F. ovata*, intra- and inter-species synteny analyses were performed on the NHX members of these three species. This revealed that whole-genome duplication (WGD) or segmental duplication served as the primary driving force for the expansion of the NHX gene family in these *Forsythia* species, contributing 80% (4/5) of the members in *F. viridissima*, 57% (4/7) in *F. ovata*, and 33% (2/6) in *F. suspensa* (S5 Table). Dispersed duplication accounted for the remaining members. Intra-species synteny analysis further identified five gene pairs (S4 Fig), all of which exhibited *K*_*a*_/*K*_*s*_ ratios significantly less than 1 (S6 Table), indicating that they have undergone strong purifying selection with conserved sequence functions. In *F. suspensa*, only one syntenic gene pair (*FsNHX3* and *FsNHX6)* was detected (S4A Fig) with an estimated divergence time of ca. 35.50 Mya. In *F. viridissima*, two syntenic gene pairs were identified (S4B Fig): For *FvNHX6* and *FvNHX4*, the divergence time is ca. 36.69 Mya, whereas *FvNHX6* and *FvNHX7* exhibit extremely large differences in their sequences, preventing the determination of an accurate *Ks* value and thus making it impossible to estimate their divergence time. Similarly, two syntenic gene pairs were detected in *F. ovata*: *FoNHX1* and *FoNHX2*, with a divergence time of 1.47 Mya, and *FoNHX4* and *FoNHX5*, with a divergence time of 0.28 Mya. These results suggest that the duplication events within *F. viridissima* occurred during a relatively recent geological period. Inter-species synteny analysis revealed eight syntenic gene pairs between *F. suspensa* and *F. viridissima*, indicating a high degree of homology and the closest genetic relationship between their NHX genes (Fig 3A). Four syntenic gene pairs were identified between *F. suspensa* and *F. ovata*, revealing less synteny than between *F. suspensa* and *F. viridissima*. Additionally, four syntenic gene pairs were identified between *F. suspensa* and *A. thaliana*, whereas only two were found between *F. suspensa* and the monocot *O. sativa*, representing the weakest syntenic relationship (Fig 3B). These findings demonstrate the extensive evolutionary conservation of the *F. suspensa* NHX gene family within congeneric species, particularly *F. viridissima* and with the dicot model plant *A. thaliana.*

**Fig 3:**
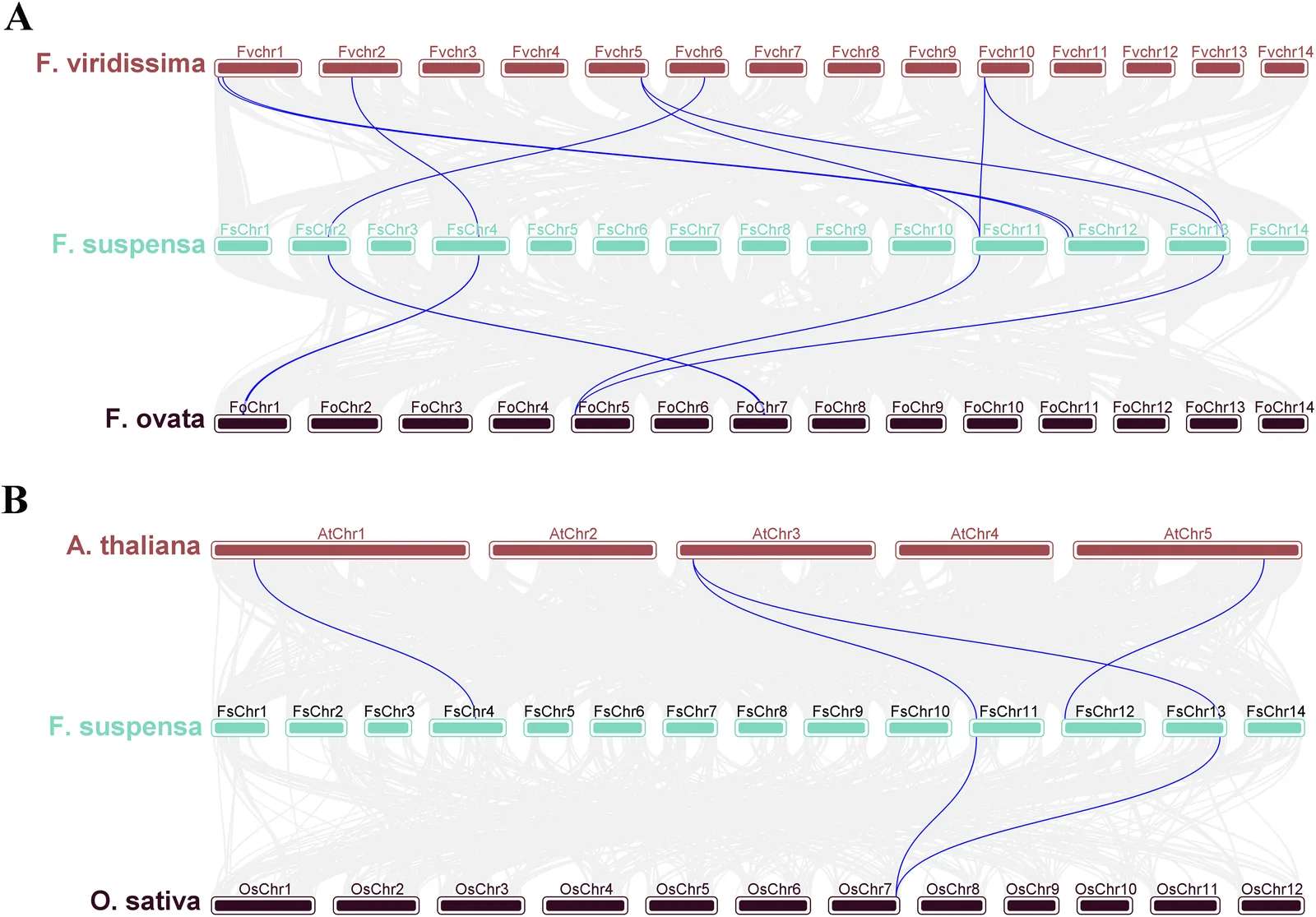
Syntenic examination of NHX genes across Forsythia and other plant species. **(A)** Syntenic examination of NHXs among *F. suspensa*, *F. viridissima*, and *F. ovata*. **(B)** Synteny comparison between *FsNHX* genes and those of *A. thaliana* and *O. sativa*. Blue lines highlight syntenic NHX homologous gene pairs; gray lines indicate collinear genomic regions.

### 3.5 Cis-element analysis

To clarify the transcriptional regulatory mechanisms of the NHX gene family in *Forsythia*, promoter sequences 2000 bp upstream of the transcription start site were retrieved for all *FsNHX*, *FvNHX* and *FoNHX* genes, and CAREs were analyzed using the PlantCARE database. Within the promoters of the 18 NHX genes, 67 different CAREs were identified. These elements were subdivided into four general functional groups: growth/developmental regulation, light, stress, and phytohormones (Fig 4; S7 Table). Of these, those related to stress were the most prevalent and widespread. The bHLH binding site (MYC) and MYB binding motifs were the most common stress-responsive elements, with 74 and 63 occurrences, respectively. The MYC motif was found in all NHX gene promoters, indicating that the transcription of this gene family may be regulated by both ABA-dependent and ABA-independent signaling pathways [15]. Anaerobic induction elements (AREs) and W box motifs, known to be involved in the response to wounding and pathogen attack, were frequent in several of the promoters, suggesting a wide spectrum of activity for this family in response to hypoxic and biotic stress signals. A wide range of light-responsive elements, such as Box 4, G-Box, and GT1-motif, were also detected, suggesting that the expression of NHX genes is finely regulated by light signals, perhaps in coordination with other light-dependent functions such as photosynthesis and photomorphogenesis. In terms of hormone–responsive elements, the core ABA responsive elements (ABREs) motifs and their variants (ABRE3a and ABRE4) were the most ubiquitous and were found in the promoters of all of the NHX genes. The CGTCA and TGACG motifs, related to responsiveness to MeJA, were often clustered together. These results reflect the importance of the ABA and jasmonate signaling pathways in controlling NHX gene expression. In contrast, elements associated with growth and development (O2-site and CAT-box) were scattered among a few genes, indicating that certain members may have specialized roles in specific tissues or developmental stages. In summary, the promoter cis‑elements of *Forsythia* NHX genes are abundant in stress‑, hormone‑, and light‑responsive elements, revealing the presence of a complex, multi‑layered transcriptional regulatory network.

**Fig 4:**
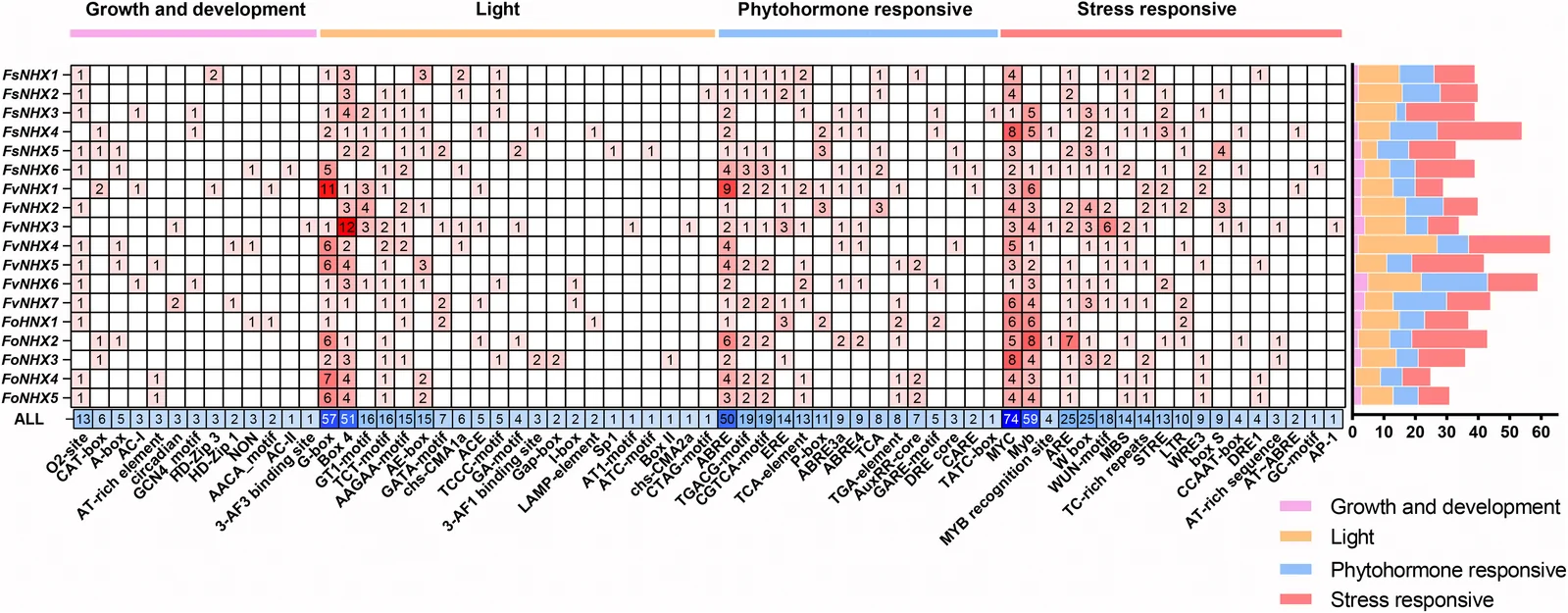
Promoter cis‑element composition of *Forsythia* NHX genes. Different colors denote distinct functional categories: pink, growth and development regulation; yellow, light; red, stress response; blue, phytohormone response; Numerical values indicate the count of each cis‑element type.

### 3.6 Transcriptome-wide expression profiling of *FsNHX* under drought and low-temperature stress

To determine whether FsNHX genes are involved in the physiological regulation of drought and cold stress and to understand their differential expression, RNA-seq data were employed to profile their expression across distinct geographic populations (S8, S9 Tables). A heat map was used to visualize their relative expression (Fig 5). In all four populations, in the leaf tissue, FsNHX5 and FsNHX6 exhibited the highest expression and FsNHX4 the lowest. Overall, the FsNHX family members exhibited markedly different responses to drought and cold stress. FsNHX3 and FsNHX6 were upregulated under both stress conditions. FsNHX5 displayed a contrasting regulatory pattern induced by drought stress but was suppressed by cold stress. FsNHX2 was broadly upregulated under drought stress, whereas significant upregulation was observed in only one specific population under cold stress. FsNHX1 and FsNHX4 were down-regulated by both treatments. These results indicate that functional divergence occurs among the FsNHX family members in F. suspensa, and the expression patterns of these genes vary among the different geographic populations.

**Fig 5:**
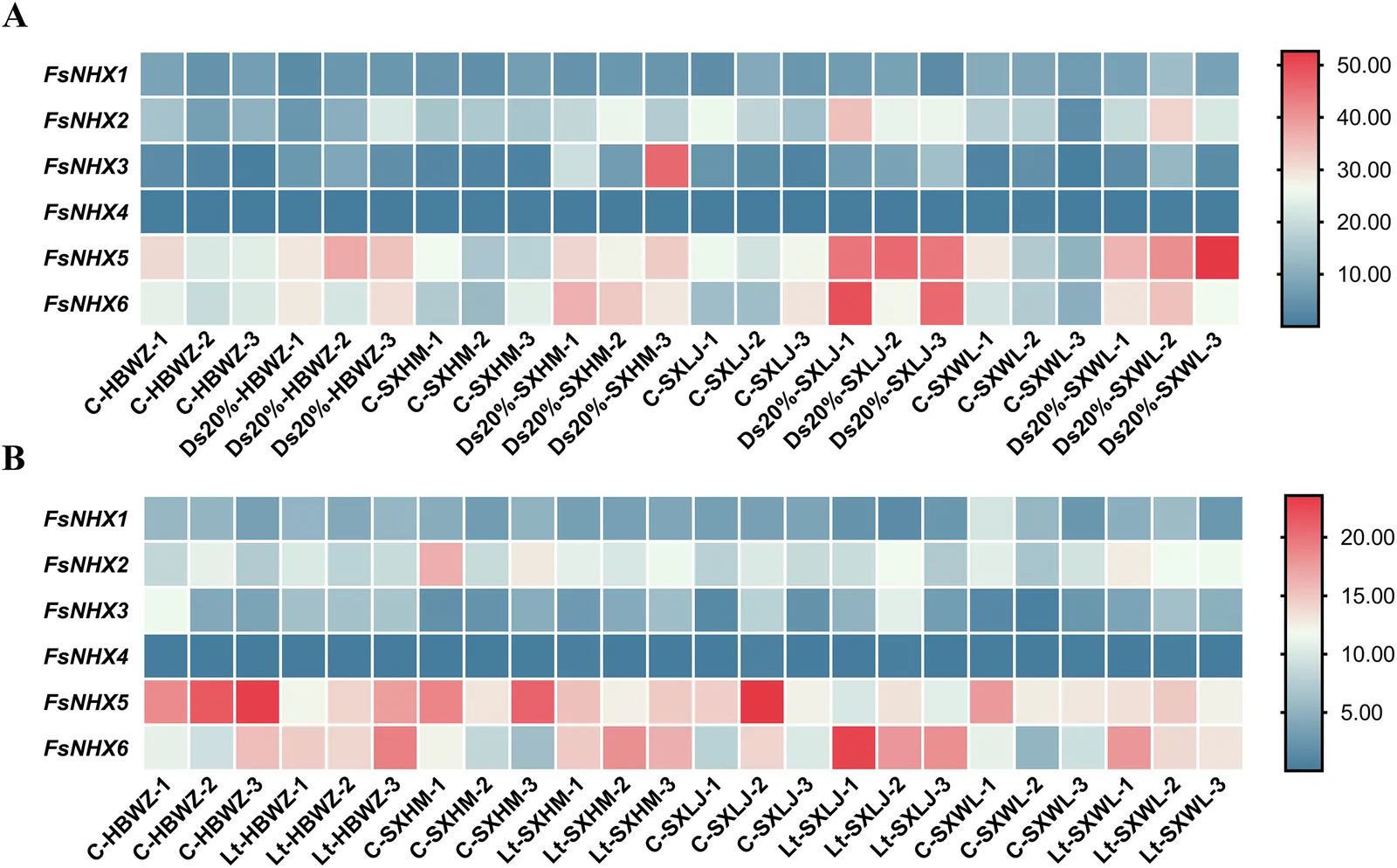
Heatmap of FsNHX expression from four populations under drought and low-temperature stress. **(A)** Expression of FsNHXs in the leaf of F. suspensa seedlings grown under soil water contents of 80% (control) and 20% for three hours. C, control; Ds20%, drought stress under soil water content of 20% for 3 hours. **(B)** Expression of FsNHXs in the leaf of F. suspensa seedlings grown at 25°C (control) and 4°C for one day. C, control; Lt, low-temperature stress at 4°C for one day. HBWZ, Wuzhi Mountain, Hebei; SXWL, Wulaofeng, Shanxi; SXHM, Huashan, Shaanxi; SXLJ, Laojun Mountain, Shaanxi. Biological replicates are labeled as 1, 2, and 3. Normalized transcripts per million (TPM) values are indicated by the color bar on the right.

### 3.7 qRT-PCR validation of FsNHX expression patterns under abiotic stress

To validate and extend the transcriptome-derived expression patterns observed under drought stress, we performed qRT-PCR analysis to examine the expression of all of the FsNHX genes under a broad set of abiotic stress conditions, including drought, salt, and ABA. This confirmed the overall trends observed in the RNA-seq data under drought stress and further revealed that all of the FsNHX genes exhibited significantly different induction patterns, with variations in response timing, intensity, and tissue specificity between the roots and leaves (Fig 6A). FsNHX3 exhibited the most pronounced upregulation in both the roots and leaves, with a more sustained induction duration (Fig 6A). Notably, in the leaves, its expression peaked 35-fold relative to the control and remained significantly higher than the control at all time points, although it declined to the level in the control at 36 h. FsNHX5 and FsNHX6 exhibited sustained upregulation in both the roots and leaves, with a gradual decline after reaching their peak or with persistent induction, suggesting their potential involvement in sustained osmotic adjustment. In the leaves, FsNHX1 and FsNHX2 were significantly upregulated only at 12 h (by 2.7- to 3.6-fold) and exhibited fluctuating expression at other time points, potentially indicating their roles in early signaling responses. In contrast, in the roots, FsNHX1 and FsNHX2 showed sustained upregulation. FsNHX4 expression in the leaves was transiently upregulated (at 12 and 96 h), but was significantly lower than that in the control at all of the other time points; in the roots, in contrast, it was strongly suppressed across the entire time course. This suggests that FsNHX4 plays a negative regulatory role under drought stress.

**Fig 6:**
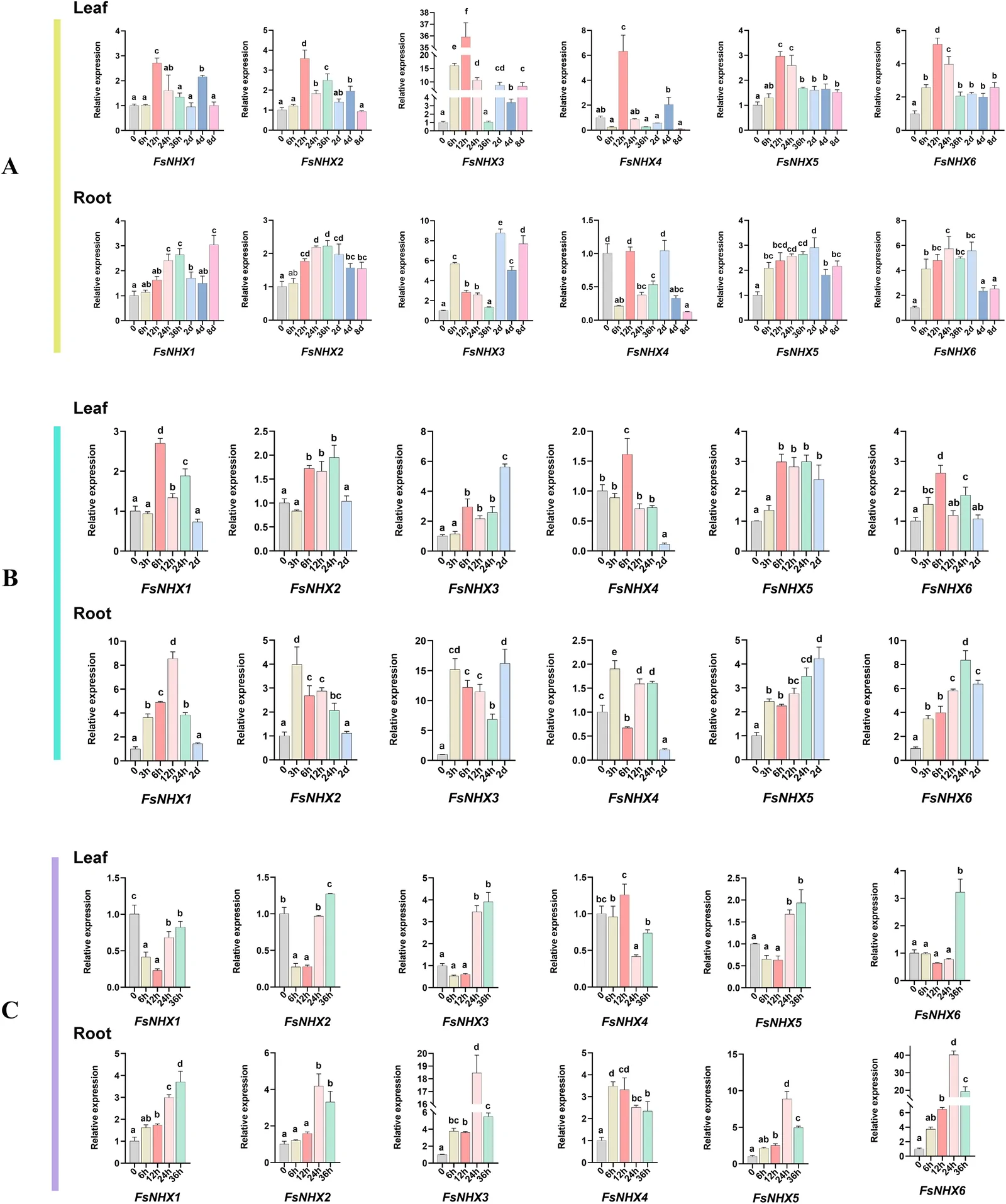
Expression profiles of *FsNHX* genes in leaves and roots in response to abiotic stress. **(A)** Expression profiles of *FsNHX* genes in leaves and roots in response to treatment with 20% PEG 6000 for 0 (control), 6 h, 12 h, 24 h, 36 h, 2 d, 4 d, and 8 d. **(B)** Transcript abundance of *FsNHX* genes in leaf and root tissues subjected to 100 mM NaCl for 0 (control), 3h, 6 h, 12 h, 24 h and 2d. **(C)** Expression profiles of *FsNHX* genes in leaves and roots with the treatment of 100 μM ABA for 0(control), 6 h, 12 h, and 24 h. The transcript levels of *FsNHX* genes were normalized against EF1αH and expressed as fold change relative to the 0 h control. Relative quantification was performed using the 2^−ΔΔCt^ method. Values represent the mean ± SD from three biological replicates. Different letters above the bars denote statistically significant differences (p < 0.05) based on one-way ANOVA with Tukey’s HSD post-hoc test.

Under salt stress, all of the *FsNHX* genes were significantly upregulated in both the roots and leaves, albeit with distinct tissue-specific patterns (Fig 6B). In the leaves, all of the *FsNHX* genes were significantly upregulated after 6 h of salt treatment. However, in the roots, the response was more rapid and stronger, with upregulation occurring as early as 3 h after salt stress. Of these, *FsNHX3* exhibited the highest expression, at ca. 14-fold higher than in the control. Under salt stress, *FsNHX3* and *FsNHX5* expression was substantially upregulated and remained at a high level even after 2 d of salt stress, indicating their significance in plant adaptation to prolonged salt stress. *FsNHX6* exhibited high and continuous expression in the roots, whereas its expression in the leaves fluctuated. Both *FsNHX1* and *FsNHX2* exhibited unimodal induction in both the root and leaf tissue, with their expression declining to control levels after 2 d of salt stress. However, the timing at which they were most expressed was different in the roots: *FsNHX1* expression peaked at 12 h, whereas *FsNHX2* expression peaked at 3 h. This suggests that *FsNHX1* and *FsNHX2* are involved in the early phase of the response to salt stress, likely participating in transient signaling, although with different roles. *FsNHX4* exhibited a unique pattern of expression: In the leaves, it was significantly upregulated only at 6 h, after which its expression declined rapidly and was significantly lower than in the control at 2 d; in contrast, in the roots, its expression fluctuated over time. The different expression profiles of the *FsNHX* genes indicate that they exhibit functional divergence under salt stress and are therefore functionally complementary.

The NHX gene family was regulated by the complex and diverse effects of ABA treatment (Fig 6C). ABA treatment consistently inhibited the expression of *FsNHX1* and *FsNHX4* in the leaves, indicating that these genes were negatively regulated by ABA stress. The expression of FsNHX2 was initially downregulated, but then increased, returning to or even surpassing the level in the control at 24–36 h. *FsNHX3*, *FsNHX5* and *FsNHX6* were all significantly upregulated at 24–36 h, with *FsNHX3* and *FsNHX6* being the most highly upregulated (by 3- to 4-fold), implying that these genes may participate in osmotic adjustment or signal transduction in response to ABA. In the roots, all of the *FsNHX* genes were strongly induced by ABA (Fig 6C). The expression of most of the genes, including that of *FsNHX3*, *FsNHX5*, *FsNHX6*, and *FsNHX2*, peaked at 24 h. *FsNHX6* exhibited the most pronounced response, with an expression level 40-fold higher than that of the control at 24 h, suggesting that it plays a key role in ABA signaling.

## 4 Discussion

The plant NHX gene family encodes Na⁺/H⁺ (or K⁺/H⁺) antiporters that mediate ion exchange across the plasma membrane, tonoplast, and endosomes. These transporters play critical roles in maintaining cellular Na^+^/K^+^ homeostasis, pH balance, osmotic adjustment, and responses to abiotic stresses such as salinity and drought [17,32,50]. In most diploid plants, the NHX gene family comprises 5–10 members, as in *A. thaliana* (eight members), *O. sativa* (seven members), *P. granatum* (ten members) [51], and *Eleusine coracana* (five members) [52]. Here, 18 NHX genes were identified in three *Forsythia* species (six in *F. suspensa*, seven in *F. viridissima* and five in *F. ovata*).

These 18 NHX proteins were grouped into three clades: Vac (12 members), PM (four members), and Endo (two members), based on phylogenetic analysis. NHX proteins from *F. suspensa* and *F. viridissima* were found in all three clades, whereas *F. ovata* did not contain any Endo-class proteins. The same result has been observed in other species, such as finger millet, in which five NHX genes have been identified and were assigned to the Vac and PM classes, with no Endo-class genes identified [52]. The lack of Endo-class NHX proteins in *F. ovata* may be due to lineage-specific gene loss or functional compensation by other NHX classes. In model species, the phylogeny of NHX proteins is broadly related to the subcellular location and biological activities of these proteins. The current findings represent an exception to this pattern: although *FsNHX5* and *FvNHX2* were placed in the Endo clade, the in silico analysis predicted them to be vacuolar proteins. Similar inconsistencies have been reported in other plants, such as pomegranate [51], tomato [53], and amaranth [54]; these inconsistencies are probably due to the limitations of the existing prediction algorithms for complex membrane proteins [55]. Therefore, the subcellular localization of *FsNHX5* and *FvNHX2* requires to experimental confirmation.

Within each of the three clades, the gene structures, numbers, and types of conserved motifs in the *Forsythia* NHX family were highly similar. In the Vac clade, most of the NHX proteins were found to contain 14 exons, those in the PM clade 17–22 exons, and those in the Endo clade 23 exons. This pattern is generally similar to that observed in *Arabidopsis*, in which proteins with plasma membrane-targeting localizations are longer, and those with intracellular-targeting localizations are shorter [7]. The same pattern has been reported for poplar [56] and tomato [53], implying that the same evolutionary constraints are at work in all dicots.

With the exception of *FoNHX1* and *FoNHX2*, which have six transmembrane domains, the other NHX proteins have 9–12 transmembrane domains, which is typical of Na^+^/H^+^ exchangers [57]. Most NHX proteins exhibit an amiloride-binding site, FFI/LY/FLLPPI, that inhibits Na^+^/H^+^ exchange activity. Although this motif was initially thought to be specific to vacuolar-type NHX proteins [58], it has been found in endosomal-type NHX proteins in many different species [53,59]. Here, interestingly, this motif was found in all Vac-class and Endo-class members; a single amino acid substitution was found in *FoNHX3* (F → V; FVIYLLPPI), whereas in the two Endo-class proteins, a single substitution (F → L; FLIYLLPPI) was observed. This finding may indicate partial functional conservation between the Vac and Endo class NHX proteins [50]. The same conserved substitutions have been described in sorghum [60]. Both residues are hydrophobic, and the overall structure of the protein and its core transport function are likely to remain the same. However, further experimental analysis is required to clarify whether this exchange influences sensitivity to inhibitors, cation selectivity, or any other regulatory properties. Importantly, this variant could be a lineage-specific trait among *Forsythia* species, and additional functional assays are required to determine whether it has any adaptive significance.

Analysis of promoter cis-acting elements revealed that the promoter regions of the *Forsythia* NHX genes contained ABRE, MYB, and MYC motifs, which are known to be involved in hormone signaling and stress responses. Therefore, these genes are likely to be widely involved in responses to abiotic stress. The results of qRT-PCR analysis of *FsNHX* expression under abiotic stress supported these findings, revealing that these genes are expressed differently in various tissues. Different members of the *FsNHX* family exhibited both transient and persistent response patterns in leaves and roots under drought and salt stress, suggesting that these *FsNHX* genes function differently and participate in different mechanisms involved in tolerance toward salt and drought stress. Vacuolar *FsNHX3* and *FsNHX6* both exhibited prolonged and strong upregulation under salt and drought stress and were also induced by ABA; thus, they can be considered ABA-dependent osmosensitive genes. Based on these findings, *FsNHX3* and *FsNHX6* may participate in Na⁺ sequestration and play an important role in K⁺ accumulation in the vacuole; their activity therefore adjusts the osmotic potential by compartmentalizing Na⁺ and K⁺ in the vacuole, enabling the plants to maintain ion homeostasis and regulate water relations at the cellular level in response to osmotic stress. *FsNHX6* exhibited the highest ABA-induced upregulation in roots. This strong induction is similar to the high expression patterns previously observed for *KvNHX1* [61] and *PbNHX2.2* [62] under ABA treatment. Thus, *FsNHX6* is likely one of the major components of the ABA signaling pathway. Based on promoter analysis, the *FsNHX6* promoter region has the highest density of ABA-responsive elements among all *FsNHX* genes. Therefore, this suggests that *FsNHX6* plays a more important role in ABA-mediated long-term osmotic adjustments under drought and salt stress than the other *FsNHX* members. In contrast, vacuolar *FsNHX1* exhibited only a short-term response in leaves under salt and drought stress and was not induced by ABA; thus, *FsNHX1* may be regulated by an ABA-independent signaling pathway. These observations are consistent with prior results for *A. thaliana* [15], rice [63], and mulberry (*Morus atropurpurea*) [64]. Therefore, it can be deduced that both ABA-dependent and ABA-independent signaling pathways exist among the *FsNHX* family members in *Forsythia*, and this is consistent with the functional predictions of their promoter elements.

*FsNHX3* was also expressed more rapidly and strongly in leaves under drought stress, but its induction in the roots under salt stress was more pronounced. Therefore, vacuolar *FsNHX* may have different physiological functions in different tissues in response to salt and drought stress. Under drought stress, the leaves are the main sites of water loss through transpiration and photosynthesis, and they are the first sites to sense water shortages. The rapid and effective induction of *FsNHX3* in the leaves enhances vacuolar K^+^/H^+^ exchange, adjusts the osmotic potential of mesophyll cells, and maintains turgor pressure, thus maintaining photosynthetic activity. The roots are the first organs to come into direct contact with high concentrations of Na^+^ under salt stress; the greater upregulation of *FsNHX3* in the roots indicates that *FsNHX3* actively transports Na^+^ into the root vacuoles to reduce the amount of Na^+^ transmitted to the shoot. This is consistent with the prior finding for *Zygophyllum xanthoxylum* that, under drought conditions, *ZxNHX* is expressed preferentially in the leaves [65]. Similarly, after 6 h of salt treatment, in *P. euphratica*, *PeNHX* exhibits far greater upregulation in the roots than in the stems and leaves [66]. Among the genes examined here, vacuolar *FsNHX3* exhibited the most pronounced and sustained transcriptional response to osmotic stress, suggesting its potential involvement in this adaptive process.

In *A. thaliana*, the endosomal proteins NHX5 and NHX6 generally exhibit an early and rapid response and are not regulated by ABA, suggesting their involvement in signal transduction or vesicle‑mediated initial Na⁺ transport [15]. However, in *F. suspensa*, endosomal *FsNHX5* was consistently upregulated under salt and drought stress and was induced by ABA. Similar expression patterns have been observed in rice endosomal *OsNHX5* [63] and grapevine endosomal *VvNHX6* [67], indicating that *FsNHX5* in *F. suspensa* possesses distinct regulatory features and may have undergone functional diversification. This further suggests that endosomal NHX members exhibit complex responses to abiotic stress. In summary, the *FsNHX* family in *F. suspensa* exhibits differential expression patterns under drought and salt stress, with members from different subclasses showing distinct temporal and tissue-specific responses. These results suggest that the coordinated yet diverse expression of *FsNHX* genes contributes to the adaptive responses of *F. suspensa* to osmotic stress.

## 5 Conclusions

The present study identifies, for the first time, all of the Na⁺/H⁺ antiporter family genes in three *Forsythia* species (*F. suspensa*, *F. viridissima* and *F. ovata*) and systematically compares these genes across the three species. Eighteen NHX members were identified and were grouped into three subfamilies: Vac, PM, and Endo. Interestingly, *F. ovata* lacked Endo-class members, which could be due to either loss of function or compensation during evolution. Synteny analysis revealed that expansion of the NHX gene family in *Forsythia* species was caused primarily by whole-genome duplication or segmental duplication. The *K*_*a*_/*K*_*s*_ ratios of all pairs of duplicated genes within each *Forsythia* species were <1, indicating strong purifying selection and a high level of conservation of the functions of these paralogs. The results of the inter-species synteny analysis reveal that *F. suspensa* and *F. viridissima* were the most similar, exhibiting the highest homology. Based on expression analysis, the *F. suspensa* NHX gene family was found to exhibit highly differentiated and tissue-specific response patterns to drought, salinity, and ABA stress. Notably, *FsNHX3* and *FsNHX6* exhibited the strongest and most sustained transcriptional induction among all of the *FsNHX* members across the three stress treatments, particularly in the roots. Given their marked responsiveness to ABA, these genes represent promising candidates for further investigation of their potential roles in ion homeostasis and osmotic adjustment under stress. Overall, this study provides valuable insights and candidate gene resources for the future functional characterization of the NHX genes involved in woody plant stress tolerance. Moreover, these findings offer potential targets for the genetic improvement of *Forsythia* varieties by enhancing their salt and drought tolerance.

## Supporting information

S1 Fig The chromosomal localization schematic for the FsNHX, FoNHX and FvNHX gene families in *Forsythia suspensa* (A), *Forsythia ovata* (B) and *Forsythia viridissima* (C). (TIF)

S2 Fig Predicted tertiary structures of NHX isoforms in *Forsythia.* (TIF)

S3 Fig Conserved domain of NHX proteins in *Forsythia.* (TIF)

S4 Fig Regional collinearity of NHX genes in three *Forsythia* Species. (TIF)

S1 Table List of primers used in RT‑qPCR. (XLSX)

S2 Table The protein, coding sequence, and promoter sequence information of FsNHXs, FvNHXs, FoNHXs retrieved from the genomes. (XLSX)

S3 Table Secondary structural makeup of *Forsythia* NHX proteins. (XLSX)

S4 Table Sequences employed for phylogenetic tree building. (XLSX)

S5 Table Analysis of duplication events within *Forsythia* NHX genes. (XLSX)

S6 Table Detailed information on the Ka, Ks and divergence-time in *Forsythia.* (XLSX)

S7 Table Full information on cis‑regulatory motifs present in NHX gene promoters. (XLSX)

S8 Table Expression of FsNHXs in leaves of *F. suspensa* seedlings grown under soil water contents of 80% (control) and 20% for three hours. (XLSX)

S9 Table Expression of FsNHXs in leaves of six-month-old *F. suspensa* seedlings grown at 25°C (control) and 4°C for one day. (XLSX)
